# Novel SEA and LG2 Agrin mutations causing congenital Myasthenic syndrome

**DOI:** 10.1186/s13023-017-0732-z

**Published:** 2017-12-19

**Authors:** Jianying Xi, Chong Yan, Wei-Wei Liu, Kai Qiao, Jie Lin, Xia Tian, Hui Wu, Jiahong Lu, Lee-Jun Wong, David Beeson, Chongbo Zhao

**Affiliations:** 1 Department of Neurology, Huashan Hospital, Fudan University, Shanghai, China; 2 Neurosciences Group, Nuffield Department of Clinical Neurosciences, Weatherall Institute of Molecular Medicine, University of Oxford, Oxford, OX3 9DS UK; 3 Baylor Genetics Laboratories, Houston, Texas USA; 4 Department of Neurology, Jing’an District Center Hospital of Shanghai, Shanghai, China; 5 Department of Molecular and Human Genetics, Baylor College of Medicine, Houston, Texas USA

**Keywords:** Congenital Myasthenic syndrome, *Agrin*, Distal myopathy, Neuromuscular junction, Salbutamol

## Abstract

**Background:**

Congenital myasthenic syndrome caused by mutations in *AGRN*, a gene encoding a protein with a crucial function at the neuromuscular junction, is a rare disorder. There are few studies in this area. We here present two cases with novel mutations of *AGRN* of which we further investigated possible pathogenesis.

**Results:**

Patient 1 had general limb weakness with fluctuation and deterioration in the afternoon and in hot weather. Patient 2 had early-onset weakness of lower extremities with suspected fluctuation in the early stages, which then progressed to the upper limbs. Both distal and proximal muscles were involved. Repetitive stimulation on EMG in both patients showed decrement in proximal and distal limbs. Patient 2 showed a marked response to salbutamol while Patient 1 did not. By targeted exome sequencing, two novel homozygous missense variants, p.L1176P and p.R1698C, in the SEA and LG2 domain of agrin were identified respectively. Further functional analysis revealed instability of the protein and impaired clustering of the acetylcholine receptor (AChR) by both mutations.

**Conclusions:**

The mutations identified in *AGRN* in our study may cause congenital myasthenic syndrome by damaging protein stability and interfering with AChR clustering. These results broaden the understandings on the phenotype, genotype and pathogenesis of this rare disorder.

**Electronic supplementary material:**

The online version of this article (10.1186/s13023-017-0732-z) contains supplementary material, which is available to authorized users.

## Background

Congenital myasthenic syndromes (CMS) are a heterogeneous group of inherited disorders characterized by impaired neuromuscular transmission, mostly resulting from genetic defects affecting neuromuscular junction (NMJ) proteins. A clinical hallmark of fatigable weakness, accompanied with abnormal jitters on single fiber electromyogram (EMG) or decrement in repetitive nerve stimulation (RNS), is suggestive of congenital myasthenia [1, 2]. Gene sequencing is necessary to establish a definitive and accurate diagnosis as it may guide appropriate therapy. To date, at least 31 different genes are known to cause CMS, which include genes encoding presynaptic proteins, postsynaptic proteins, components of the synaptic basal lamina, proteins related to endplate development and maintenance, and more recently proteins involving glycosylation [3]. Here we present two cases of an uncommon form of CMS with different homozygous missense mutations in AGRN.

## Methods

### Patients

Thirty patients were clinically and electrophysiologically diagnosed as CMS in Huashan Hospital during 2009–2016. Among them, 2 patients harboring homozygous *AGRN* variants were enrolled in this study. Detailed clinical information was collected. Written informed consent was obtained for genetic analysis and publication. This study was approved by the Huashan Hospital (Fudan University) Institutional Review Board.

### Molecular studies

Genomic DNA from blood was extracted with High Pure PCR Template Preparation Kit (Roche,Basel, CH) according to the manufacturer’s instructions. For patient 1, 17 genes (*AGRN, ALG14, ALG2, CHAT, CHRNA1, CHRNB1, CHRND, CHRNE, COLQ, DOK7, DPAGT1, GFPT1, LAMB2, MUSK, PLEC, RAPSN, SCN4A*) known to cause CMS were enriched using target capture (Baylor Genetic Laboratories, Houston, Texas, USA) and subjected to sequencing on Illumina HiSeq2000 [4]. For patient 2, a commercial next generation sequencing (NGS) panel was used (PrecisionMD, China, including *AGRN, ALG2, ALG14, CHAT, CHRNA1, CHRNB1, CHRND, CHRNE, COLQ, DOK7, DPAGT1, GFPT1, LAMB2, MUSK, RAPSN* and *SCN4A*) and subsequent sequencing was conducted on the Illumina MiSeq. References to nucleotides or amino acids are based upon the genomic DNA (NC_000001.11) and cDNA (NM_198576) sequence for *AGRN*. SIFT and PolyPhen-2 were used to predict the pathogenicity of novel missense variants.

### Functional assays

To explore the effect of two novel variants, they were respectively introduced into cDNA by site-directed mutagenesis using Quickchange kit (Stratagene, USA). HEK 293 cells were transfected with 18 μg pDNA3.1hygro(+) GFP-tagged wild type and mutant agrin. The recombinant protein contains its own signal peptide, which allows agrin to be secreted as a soluble form. pDsRed-monomer-N1 was co-transfected to verify transfection efficiency. Forty eight h following transfection, whole cell lysates and conditioned media of HEK 293 cells transfected with either wild type or mutant agrin were harvested. The level of agrin expression was detected by western blot using mouse anti-GFP antibody (ad6556, Abcam), HRP-conjugated anti-mouse secondary antibody (Dako) and ECL (GM Healthcare). DesRed was used as a marker to verify transfection efficiency and alpha-tubulin in total cell lysates served as a loading control. Densitometry of protein bands at ~250KD in conditioned media, which corresponds to the translated ‘full length’ cDNA transcript with the GFP tag, was analyzed using ImageJ software. We further determined turnover or half-life of full length wild type and mutant agrin. We treated the transfected cells with cycloheximide (20 μg/ml) to block further protein synthesis, then the media were collected at a series of time points and the level of agrin was determined by western blotting as described above.

C2C12 myotubes were also exposed to the same amount of wild type or mutant agrin-containing medium for 16 h, Medium from non-transfected HEK293 cells was set as a control. The cells were incubated with α-Butx-594(Invitrogen, USA). Images (20 random fields at 20× objective) were captured using an Olympus IX71 fluorescence microscope with Simple PCI (Digital Pixel). Size and number of AChR clusters were analyzed using the ImageJ Macro automated counting system. The size cut-off for a cluster is 2.5 μm^2^.

### Statistical analysis

Statistical analysis was performed using GraphPad Prism. For expression of full-length mutant and wild type agrin, statistical comparison was performed by two-way ANOVA with multiple comparisons. For AChR clustering assay, data was analyzed using unpaired Student’s t-test. *P* value was considered to be significant when *p* < 0.05.

## Results

### Clinical presentation

**Fig. 1:**
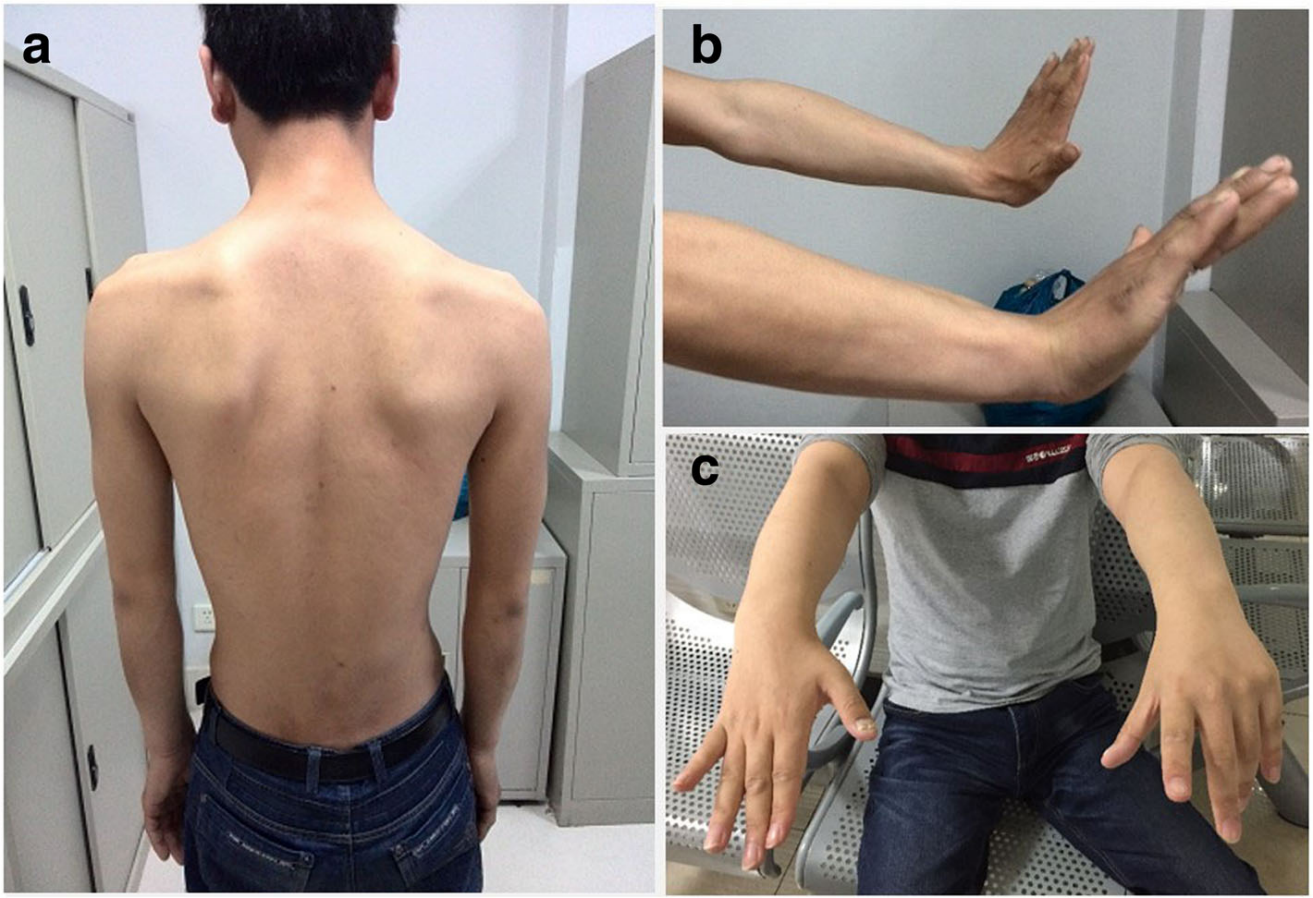
**a** Patient 1 showed limb girdle weakness with bilateral scapular winging. **b**, **c** Distal involvement was both seen in patient 1 and patient 2

Patient 1 is a 28-year-old male born from a consanguineous family, complaining of limb weakness for 12 years. There was no learning problems. He presented with weakness of both proximal and distal extremities with a clear fluctuation and noted deterioration in the afternoon and in hot weather. When he was firstly seen by us at the age of 26, examination revealed bilateral scapular winging and pronounced atrophy of shoulder girdle muscles with involvement of distal limbs (Fig. 1a, b). Ocular, facial, bulbar, respiratory and neck muscles were not involved. Muscle strength tests showed marked limb girdle weakness (MRC grade 3–4/5), arms more affected than legs and distal muscles more than the proximal. MRI of lower limbs showed mild fatty infiltration without selective involvement or significant muscle atrophy (Additional file 1: Figure S1). During the study of nerve conduction, normal compound muscle action potentials (CMAPs) were recorded. Repetitive nerve stimulation revealed 38% and 20% decrement of CMAP in trapezius and abductor policis brevis (APB) at 3 Hz, respectively, without increment at 30 Hz stimulation. Post-exercise facilitation (PEF) was not found. EMG showed myopathic changes. There were no specific clues for a diagnosis from a muscle biopsy (biceps brachii) except varied fiber size. He did not show a response to anticholinesterase inhibitors and salbutamol.

Patient 2 is a 23-year-old male, the only child of consanguineous parents with a negative family history. He has normal motor milestone and learning abilities. Weakness of lower extremities was first noted at the age of 11. The symptom worsened with fluctuation in the following 3 years. At the age of 14, he had a difficulty in climbing stairs, and presented with upper limb weakness. These symptoms worsened progressively. When the patient was first seen by us at the age of 20, he was non-ambulant and unable to raise his arms. On clinical examination he had marked symmetric weakness of four limbs without ocular, facial, bulbar, respiratory and neck weakness involvement. Distal and proximal muscles were both affected to a similar degree (MRC grade 3/5). Bilateral Hoffmann signs were positive and hyperreflexia was detected throughout. MRI of neck and thoracic region was normal. Similar findings to Patient 1 were shown according to MRI of lower limbs. Normal CMAPs were recorded. Repetitive stimulation of median, accessory and peroneal nerve revealed clear decrements ranged from 14% to 34%, while 30 Hz stimuli didn’t elicit potentiation. No PEF was recorded. EMG also exhibited myopathic changes. A biopsy of biceps brachii was then performed and showed variation of fiber size. Based on the early-onset weakness with a history of fluctuation and decrements in RNS testing, a diagnosis of CMS was considered. Initially pyridostigmine was added but the patient showed little response. When salbutamol was administered, the symptoms improved markedly so that he regained ambulation 3 months later. However, he still had weakness in distal limbs (Fig. 1c).

### Genetic analysis

The genetic analysis in Patient 1 revealed a novel homozygous variant (c.3527 T>C) located in exon 21 of AGRN, leading to the substitution of a well-conserved leucine to a proline in the sperm protein, enterokinase and agrin (SEA) domain (p.L1176P) (Additional file 1: Figure. S3). This variant was predicted to be damaging by SIFT and PolyPhen-2, respectively (Additional file 1: Table S1). The mutation was confirmed in Patient 1 and also found heterozygous in his parents by Sanger sequencing (Additional file 1: Figure S2).

Targeted next generation sequencing panel analysis of Patient 2 revealed a homozygous missense variant in AGRN (c.5092C > T, p.R1698C), which was later confirmed in his parents (Additional file 1: Figure S2). A heterozygous missense variant, c.117C > G, (p.N39 K) in CHRND was also detected (Additional file 1: Table S1). No repetitive CMAP from a single nerve stimulus was documented in Patient 2 and the variant was also found in his asymptomatic father (Additional file 1: Figure S2), so we did not think the patient was a slow channel syndrome case cause by a heterozygous mutation in CHRND. We also ruled out CNV (copy number variation) in CHRND [5]. The arginine residue is not fully conserved across species (Additional file 1: Figure S3), while the p.R1698C substitution in the C-terminal laminin G-like (LG) 2 domain of *AGRN* was predicted to be possibly damaging by Polyphen-2 and damaging by SIFT (Additional file 1: Table S1). In this case, we considered it highly likely to be the disease-causing mutation.

### Mutations in *AGRN* caused instability of the protein and impaired ability to induce AChR clustering

**Fig. 2:**
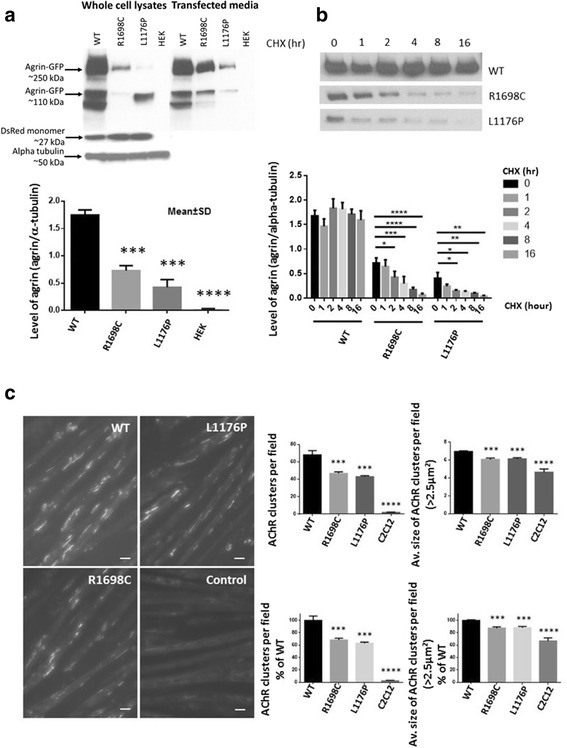
**a** Western blot analysis in whole cell lysates and media of HEK293 cells 48 h after transfection with wild type, R1698C or L1176P agrin. Levels of full length agrin (~250KDa) in the media were compared in the bar chart, *n* = 3. Alpha tubulin (~50KDa) was used as a loading control for cell lysates and DsRed monomer (~27 kDa) as a marker to verify transfection efficiency; (**b**) 48 h post HEK293 cell-transfection cells were incubated in cycloheximide (CHX) (20 μg/ml) for the indicated times above the collumns, the mutant agrin showed a time-course degradation not seen for wild type, n = 3; (**c**) Representative image of myotubes labelled with α-Butx-594 exposed to either wild type, R1698C, L1176P agrin for 16 h in media containing an equivalent agrin concentration. Magnification: 20×. The scale bar represents 10 μm. Bar charts showing the number and average size of AChR clusters per field, n = 3. (Data represents the mean ± SD of three experiments. * *p* < 0.05, ** *p* < 0.01, *** *p* < 0.001, **** *p* < 0.0001)

As shown in Fig. 2a, the expression level of full length mutant agrin both in conditioned media and whole cell lysates detected on Western blot (~250 kDa) was reduced comparing with wild type (Fig. 2a). Clearly the environment within HEK293 cells are not the same as the terminal bouton of a motor nerve, but we only statistically compare the expression of full length mutant and wild type agrin in the medium.

We also investigated the stability of wild type and mutant agrin. The rate of degradation of the protein was analized by treating transfected HEK293 cells with cycloheximide (20 μg/ml) 48 h after transfection [5]. The media were collected at a series of time points and the level of agrin was also determined by western blotting. The result showed a time-course of degradation of mutant agrin not seen for wild type (Fig. 2b).

We then evaluated the ability of mutant and wild type agrin to induce clustering of AChR. C2C12 myotubes were exposed to medium containing equivalent concentration of mutant and wild type agrin judged by western blot for 16 h. The number and size of the AChR clusters was reduced per field compared to wild type, indicating an impaired ability to induce clustering of AChR by mutant agrin (Fig. 2c).

## Discussion

Agrin is a heparan sulfate proteoglycan that occurs in multiple tissues as different isoforms generated by alternative splicing with diverse functions [6]. Motoneuron-derived agrin is considered to play an indispensable role in the formation and maintenance of NMJ [7, 8]. The protein binds to laminin via its N-terminal agrin (NtA) domains [9] and interacts with α-dystroglycan [10] and low-density lipoprotein receptor-related protein 4 (LRP4) through its C-terminal end of LG domains [11]. Two specific splice variant amino acid inserts of neural agrin at the C-terminal LG2 and LG3 domain respectively, called A and B in chickens [12] or y and z in humans [13], are required for interactions [14].

To date, 12 cases of CMS due to mutations of *AGRN* have been reported [15–19] and the mutations are distributed in LG2, LG3, NtA and follistatin-like domains (Additional file 1: Figure S4). Here we report the identification of two CMS patients carrying novel mutations in SEA and LG2 domains in agrin that further define disease-causing mutations for this disorder.

According to previously reported cases, CMS due to *AGRN* mutations may show prominent distal muscle weakness and atrophy [16]. Our patients shared several common features with five previously reported CMS patients reminiscent of distal myopathies, including marked distal weakness affecting initially the lower and later the upper limbs, sparing of axial and oculobulbar muscles and no beneficial effect to acetylcholinesterase inhibitors (Table 1). However, reduced CMAP at rest and an incremental CMAP following exercise in the previously reported 5 patients [16], suggesting presynaptic abnormality [3], was not found in our patients and other reported cases with *AGRN* mutations. More neurophysiological studies are needed to clarify this situation.

**Table 1:** Clinical features of reported *AGRN*-mutant CMS

Reports	Gender	Onset	Fluctuation	Clinical forms	RNS	Mutations	Affected domain	Response to Treatment		
				Ocular/Facial/Proximal/Distal/Respiratory	(3 Hz)			AChEI	3,4-DAP	β2-receptor agonist
2009 [15]	F	Early childhood	+^a^	+/+/+/+/ND	+	G1709R	LG2	–	–	+
						G1709R				
2009 [15]	M	Early childhood	+	+/ND/+/ND/ND	+	G1709R	LG2	–	+	+
						G1709R				
2012 [17]	F	Early childhood	+	+/+/+/+/+	+	V1727F	LG2	+	–	–
						Q353X	FS			
2014[16]	F	15y	–	−/−/+/+/ND	+	G76S	NtA	–	ND	ND
						chr1del	chr1del			
2014[16]	M	15y	–	−/−/−/+/ND	+	G76S	NtA	–	ND	ND
						chr1del	chr1del			
2014[16]	M	2y	+	−/−/+/+/−	+	N105I	NtA	–	–	+
						S455Q	FS			
2014[16]	F	At birth	+	−/+/ND/+/−	+	N105I	NtA	–	–	+
						S455Q	FS			
2014[16]	M	5y	–	+/ND/+/+/ND	+	G1871R	LG3	ND	ND	+
						G1871R				
2017 [18]	M	1.5y	ND	+/ND/+/−/ND	+	G1675S	LG2	+	ND	+
						G1675S				
2017[19]	M	21y	+	−/−/+/+/−	+	A1768P	LG2	+/−^b^	ND	+
						A1768P				
2017[19]	F	7y	ND	−/−/+/+/−	ND	A1768P	LG2	+/−^b^	ND	+
						A1768P				
2017[19]	F	ND	ND	ND	ND	A1768P	LG2	+/−^b^	ND	+
						A1768P				
P1	M	16y	+	−/−/+/+/−	+	L1176P	SEA	–	ND	–
P2	M	9y	+	−/−/+/+/−	+	R1698C	LG2	–	ND	+

*M* male, *F* female, *y* years old, *AChEI* acetylcholinesterase inhibitor, *3,4-DAP* 3,4-diaminopyridine, *ND* not determined, ^a^worsened during periods and pregnancy, *P1* patient 1, *P2* patient 2, *chr1del* large deletion covering entire *AGRN* gene, *FS* follistatin-like domain, ^b^beneficial response during the 1st month, but then symptoms aggravated

Four mutations in LG2 domain, pG1675S, p.G1709R, p.V1727F and p.A1768P, have been reported in 6 patients [15, 17–19]. Two patients presented with ptosis and general limb weakness, one isolated case manifested with proximal weakness and head drop, and a recently reported family developed proximal and distal weakness. In our study, Patient 2 showed predominant distal weakness and atrophy. All cases showed varying responses to salbutamol (Table 1). Based on previous studies, the LG2 domain has a critical role in the activation of the LRP4-MuSK complex, as neural agrin induces MuSK phosphorylation by interacting with LRP4 via its LG2 domain and then triggers the aggregation of AChR in the postsynaptic membrane [20]. Heparin, as well as several monoclonal antibodies, could block agrin-induced MuSK activation and AChR aggregation by binding to the LG2 domain [21–23]. In addition, the LG2 domain also participates into the structuring of the basal lamina through its interaction with α-dystroglycan [24]. Existing functional analysis of p.G1709R and p.V1727F showed different results (Additional file 1: Figure S4). The mutation, p.G1709R, in chicken mini-agrin did not reduce the activation of muscle-specific tyrosine kinase (MuSK) or affect the binding of agrin to α-dystroglycan, indicating that the mutant protein does not interfere with the induction of the postsynaptic apparatus but disturbs the maintenance of the NMJ (Additional file 1: Figure S4). While in the neural form of full length agrin, p.R1698C mutant agrin found in Patient 2, in consistent with the reported p.V1727F mutant protein, exhibited impaired ability to induce AChR clustering [16]. We also found the p.R1698C protein degraded faster than the wild type, which predicts that the stability of the protein is impaired. The specific molecular mechanism of phenotype difference and pathogenicity of the mutations remains to be explored.

The SEA (Sperm protein, Enterokinase and Agrin) domain named after the first three proteins in which it was identified, located in the middle of agrin, is a poorly characterized protein motif found in extracellular matrix associated glycoproteins. Recombinant agrin protein without an SEA domain could achieve similar potency in AChR clustering as seen in full-length constructs [20] and rescued *AGRN*-knockout mice [25]. It was not implicated as important for agrin function until the identification of an *AGRN* mutant (nmf380-F1061S) mouse model of CMS. NMJs in the homozygous mutant mice progressively degrade postnatally and have decreased acetylcholine receptor density (Additional file 1: Figure S4). Intriguingly, substitution of Leucine by Proline found in Pt 1 (p.L1176P) is an amino acid next to the mutant point in the mouse model, which is located at the C-terminal of an alpha-helix by a structural prediction model extrapolated from the structure of Mucin16 SEA domain [26]. Both amino acids were conserved not only among species but also SEA domains in other proteins like mucin and enterokinase. In the mouse model, the mutation did not alter the expression but impaired conformation and secretion of the protein [27]. While as shown in our results, the expression of p.L1176P mutant agrin was reduced both in whole cell lysates and media of transfected HEK293 cells. Thus, we believe both the export of the agrin into the medium and its stability in the medium are affected. However, whether this arose through altered glycosylation resulting in abnormal trafficking to the membrane for secretion or protein misfolding leading to altered stability requires further elucidation.

In conclusion, we identify two cases of *AGRN*-CMS due to homozygous mutations in the LG2 and SEA domain of agrin. Functional analysis suggested impaired stability of the mutant agrin in the conditioned medium and also probably within cellular environment of the nerve, and this is likely to be the main molecular pathogenic mechanism of the mutations for these two patients.

## Additional file

Additional file 1: Table S1. Variants found in two patients (c.117C > G in *CHRND* has been seen in ExAC 649 times and was classified in ClinVar as benign variant by Emory Genetics and Prevention Genetics. It should be a common SNP, not mutation no matter the inheritance is AD or AR). **Figure S1.** MRI of lower limbs in 2 patients showed mild non-selective fatty infiltration without significant proximal and distal muscle atrophy. **Figure S2.** Sanger sequencing of variants found in two patients and their parents. **Figure S3.** Sequence alignment of human agrin with the other species. Multiple sequence alignments were performed by uniprot (http://www.uniprot.org, May 3rd, 2017). **Figure S4.** Schematic representation of agrin with the positions and functional studies of all reported mutations to date. SS: signal sequence, LE: laminin EGF-like domain, S/T: serine/threonine-rich glycosaminoglycan attachment site, EG: EGF-like domain. See the references in the article. (DOCX 563 kb)

## Abbreviations

AChR: acetylcholine receptor
AGRN: agrin
APB: abductor policis brevis
CMAP: compound muscle action potential
CMS: congenital myasthenic syndrome
CNV: copy number variation
DMEM: Dulbecco-modified essential medium
EMG: electromyogram
LG: laminin G-like
LRP4: lipoprotein receptor-related protein 4
MuSK: muscle-specific tyrosine kinase
NMJ: neuromuscular junction
NtA: N-terminal agrin
PEF: post-exercise facilitation
RNS: repetitive nerve stimulation
SEA: Sperm protein, Enterokinase and Agrin
