# Supplementary material for: Novel SEA and LG2 Agrin mutations causing congenital Myasthenic syndrome
Source: Orphanet J Rare Dis. 2017 Dec 19;12:182. doi: 10.1186/s13023-017-0732-z (PMC5735900; doi:10.1186/s13023-017-0732-z)
Supplement: Additional file 1: Table S1. — Variants found in two patients (c.117C > G in CHRND has been seen in ExAC 649 times and was classified in ClinVar as benign variant by Emory Genetics and Prevention Genetics. It should be a common SNP, not mutation no matter the inheritance is AD or AR). Figure S1. MRI of lower limbs in 2 patients showed mild non-selective fatty infiltration without significant proximal and distal muscle atrophy. Figure S2. Sanger sequencing of variants found in two patients and their parents. Figure S3. Sequence alignment of human agrin with the other species. Multiple sequence alignments were performed by uniprot (http://www.uniprot.org, May 3rd, 2017). Figure S4. Schematic representation of agrin with the positions and functional studies of all reported mutations to date. SS: signal sequence, LE: laminin EGF-like domain, S/T: serine/threonine-rich glycosaminoglycan attachment site, EG: EGF-like domain. See the references in the article. (DOCX 563 kb) [file 13023_2017_732_MOESM1_ESM.docx]

**Supplementary Data**

**Table S1** Variants found in two patients (c.117C>G in *CHRND* has been seen in ExAC 649 times and was classified in ClinVar as benign variant by Emory Genetics and Prevention Genetics. It should be a common SNP, not mutation no matter the inheritance is AD or AR)

| **patient** | **Gene** | **cDNA** | **Amino**  **Acid** | **zygosity** | **HGMD** | **SNP** | **Allele**  **Frequency** | **Polyphen-2** | | **SIFT** | | **ExAC**  **(Allele count/Total alleles )** |
| --- | --- | --- | --- | --- | --- | --- | --- | --- | --- | --- | --- | --- |
|  |  |  |  |  |  |  |  | **HumVar** | **prediction** | **sift score** | **prediction** |  |
| 1 | *AGRN* | c.3527T>C | L1176P | homo | No | novel | Novel | 0.995 | PROBABLY  DAMAGING | 0.00 | DAMAGING | Novel |
| 2 | *AGRN* | c.5092C>T | R1698C | homo | No | rs771576402 | Novel | 0.698 | POSSIBLY  DAMAGING | 0.020 | DAMAGING | 1/117946 |
| 2 | *CHRND* | c.117C>G | N39K | het | No | [rs77084550](http://www.ncbi.nlm.nih.gov/projects/SNP/snp_ref.cgi?rs=rs77084550) | 0.007 | 0.012 | BENIGN | 0.027 | DAMAGING | 649/120600 |

homo: homozygous; het: heterozygous; Allele Frequency was obtained from 1000 Genomes(<https://www.ncbi.nlm.nih.gov/projects/SNP>). ExAC: <http://exac.broadinstitute.org>; Polyphen-2: <http://genetics.bwh.harvard.edu/pph2>; SIFT: <http://sift.jcvi.org/www/SIFT_seq_submit2.html>. All databases were accessed on May 4^th^, 2017. Polyphen-2<0.15: benign, 0.15-0.85: possibly damaging, >0.85: probably damaging; SIFT<=0.05: damaging, >0.05: tolerated.

All references to nucleotides or amino acids are based upon the genomic DNA (NC_000001.11) and cDNA (NM_198576) sequence for *AGRN* as well as the genomic DNA (NC_000002.12) and cDNA (NM_000751) sequence for *CHRND.*


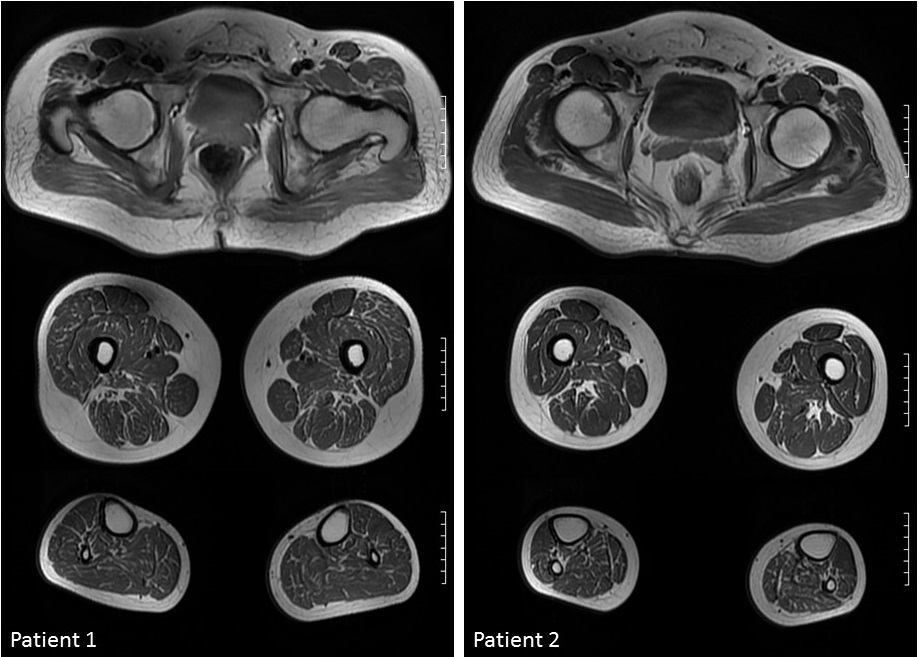


**Figure S1** MRI of lower limbs in 2 patients showed mild non-selective fatty infiltration without significant proximal and distal muscle atrophy.


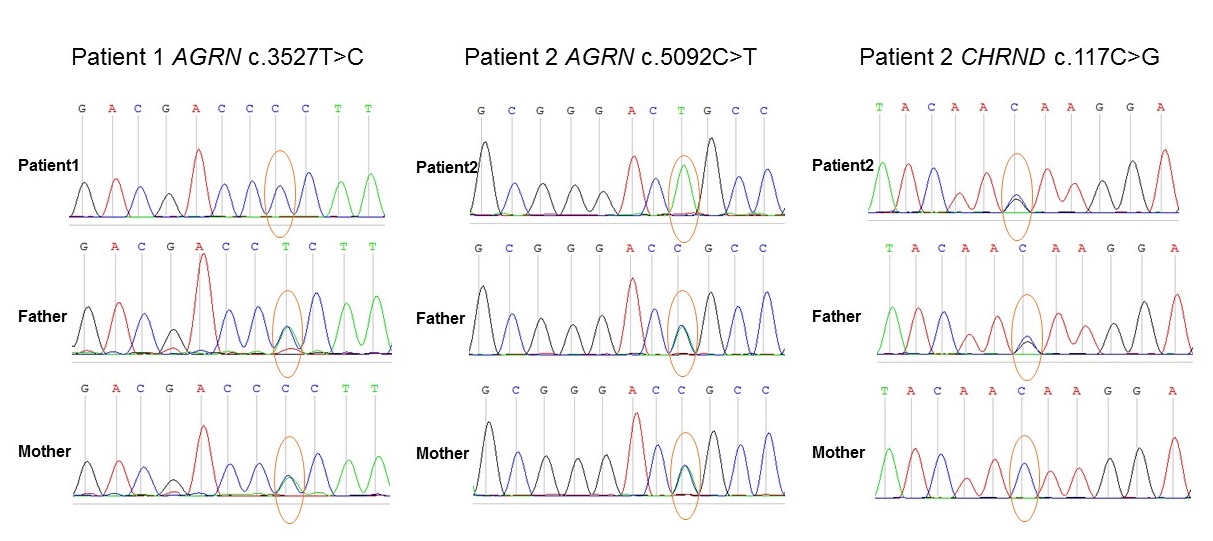


**Figure S~~1~~2** Sanger sequencing of variants found in two patients and their parents.


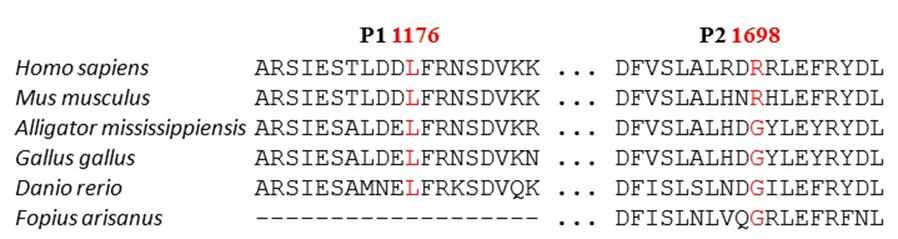


**Figure S~~2~~3** Sequence alignment of human agrin with the other species. Multiple sequence alignments were performed by uniprot (<http://www.uniprot.org>, May 3^rd^,2017)


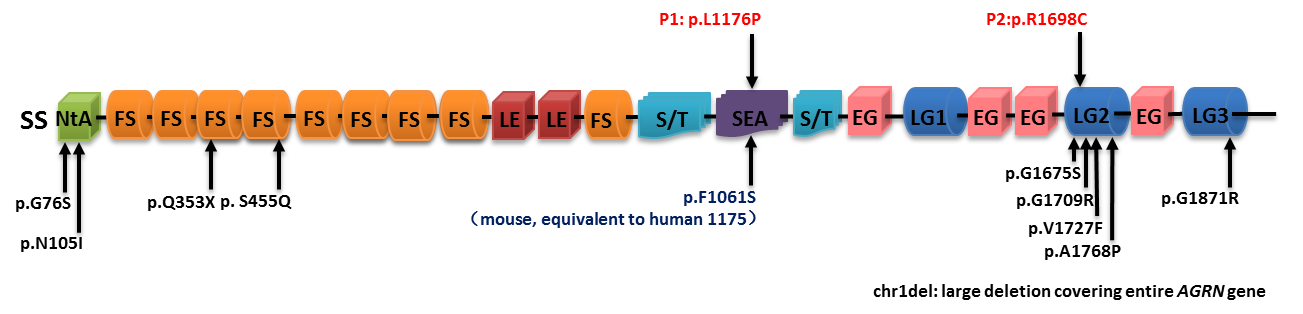


| **Affected Domain** | **Mutations** | **Clinical forms** | **Model** | **Functional analysis** |
| --- | --- | --- | --- | --- |
|  |  | Ocular/Facial/Proximal/Distal/Respiratory |  |  |
| NtA | p.G76S[16] | -/-/-/+/ND | In vitro-full-length mouse agrin cultured with C2C12 myotubes | Reduced AChR clustering |
|  | p.N105I[16] | -/-/+/+/- | Same with “p.G76S” | Same with “p.G76S” |
| FS | p.Q353X[17] | +/+/+/+/+ | Patient muscle-biopsy specimen | Disrupted NMJ architecture |
|  |  |  | In vitro-full-length rat agrin | Abolished expression of full-length agrin |
|  | p.S455Q[16] | -/+/ND/+/- | Not evaluated | Not evaluated |
| SEA | p.L1176P(P1) | -/-/+/+/- | In vitro-human agrin cultured with C2C12 myotubes | Accelerated degradation of mutant protein  Impaired AChR clustering |
|  | p.F1061S[27] | ND/ND/ND/ND/ND | Mouse model | Mutant protein: decreased glycosylation, less efficient externalization and secretion  Decreased AChR density and increased subsynaptic reticulum |
| LG2 | p.G1675S[18] | +/ND/+/-/ND | Not evaluated | Not evaluated |
|  | p.R1698C(P2) | -/-/+/+/- | Same with “p.L1176P” | Same with “p.L1176P” |
|  | p.G1709R[15] | +/ND/+/ND/ND | Patient muscle-biopsy specimen | Perturbed NMJ structures (denervated, remodeling, neoformed)  Normal expression and localization of agrin and MuSK |
|  |  |  | In vitro-chicken mini-agrin cultured with C2C12 myotubes and HEK293T cells expressing LRP4 and MuSK | Normal AChR clustering at the postsynaptic membrane  Normal MuSK phosphorylation in nonmuscle cells expressing LRP4 and MuSK  Normal binding to α-dystroglycan |
|  |  |  | In vivo-chicken mini-agrin injected into rat soleus muscle | Perturbed NMJ structures  Normal AChR clustering |
|  | p.V1727F[17] | +/+/+/+/+ | Patient muscle-biopsy specimen | Same with “p.Q353X” |
|  |  |  | In vitro-full-length rat agrin cultured with C2C12 myotubes and Immunoprecipitation | Decreased phosphorylation of MuSK and AChR β subunit  Increased binding to α-dystroglycan  Decreased binding to a z site-specific antibody  Decreased AChR clustering |
|  | p.A1768P[19] | -/-/+/+/- | Not evaluated | Not evaluated |
| LG3 | p.G1871R[16] | +/ND/+/+/ND | Not evaluated | Not evaluated |
| Entire gene | chr1del[16] | -/-/+/+/ND | Not evaluated | Not evaluated |

**Figure S4** Schematic representation of agrin with the positions and functional studies of all reported mutations to date. SS: signal sequence, LE: laminin EGF-like domain, S/T: serine/threonine-rich glycosaminoglycan attachment site, EG: EGF-like domain. See the references in the article.

**Material and Methods for functional Analysis**

1. **Cell culture**

HEK 293 cells purchased from ATCC were seeded at 3.5 x 10^6^ cells per T125 tissue culture flask in 5% CO_2_ at 37°C in Dulbecco-modified essential medium (DMEM) (Sigma-Aldrich) supplemented with 10% FCS (TCS Cellworks Ltd, UK) and 100 units/ml each of penicillin G and streptomycin (PS) (Invitrogen, USA).

1. **Production of wild-type and mutant agrin recombinant proteins**

cDNA encoding the full length human neural agrin, containing the 4- (KSRK) and 8- (ELANEIPV ) amino acid inserts at the y site and z sites, was cloned into pcDNA3.1hygro(+). A GFP-tag was also attached at the C-terminus of the agrin. The recombinant protein contains its own signal peptide, which allows agrin to be secreted as a soluble form. The introduction of R1698C and L1176P were performed by site-directed mutagenesis using Quikchange kit (Stratagene, USA). The correct reading frame of the agrin sequences were confirmed by DNA sequencing.

1. **Expression of soluble agrin**

HEK 293 cells were transfected with 18μg pDNA3.1hygro(+) GFP-tagged wild type and mutant agrin. pDsRed-monomer-N1 was co-transfected to verify transfection efficiency. Following 48 hours of transfection, ~~Following 48 hours of transfection,~~ whole cell lysates and conditioned media of HEK 293 cells transfected with either wild type or mutant agrin were harvested, respectively. The level of agrin expression was detected using mouse anti-GFP antibody (ad6556, Abcam), HRP-conjugated anti-mouse secondary antibody (Dako) and ECL (GM Healthcare). DesRed was used as a marker to verify transfection efficiency and alpha-tubulin in total cell lysates as a loading control. Experiments were carried out 3 times. Densitometry of bands at ~250KD in conditioned media, which corresponds to the intact ‘full length’ cDNA transcript with the GFP tag, was analyzed using ImageJ software and the protein expression indicated as agrin:α-tubulin was then quantitated.

We further determined turnover or half-life of wild type and mutant agrin. HEK293 cells (flask) were first transfected with 18μg pDNA3.1hygro(+) GFP-tagged wild type and mutant agrin using polyethyleneimine. Then following 48 hours after transfection, either wild type or mutant agrin being synthesized and exported into the conditioned media，the transfected cells were treated with cycloheximide (20 μg/ml) in a time-course experiment to block further protein synthesis. The media were collected at a series of time points and the level of agrin was detected by western blotting using a mouse anti-GFP antibody (ad6556, Abcam) to show differences in protein half-life in the media.

1. **Acetylcholine Receptor Clustering Assay**

C2C12 cells were seeded at 6 x 10^4^ per well in 24-well plates and were allowed to fuse to form myotubes. C2C12 myotubes were exposed to agrin contained medium for 16 hours typically diluted at 1:100 at 37°C. The amount of mutant agrin proteins in the conditioned medium from transfected HEK293 cells was concentrated using Microcon-30 centrifugal filters (Millipore) to a similar level as wild-type. The level of mutant agrin proteins as judged by western blot to be similar to that of wild type protein was used. C2C12 myotubes were exposed to medium from non-transfected HEK293 cells as a control. The following day cells were incubated with α-Butx-594 diluted at 1:1000 in fusion medium for 1 hour at 37oC. Cells were washed 3 times in fusion medium, fixed with 3% formaldehyde, and stored in PBS at 4°C. Samples were analyzed blinded, and images (20 random fields at 20x objective) were captured using Olympus IX71 fluorescence microscope with Simple PCI (Digital Pixel). Size and number of AChR clusters were analyzed using the ImageJ Macro automated counting system after setting the background threshold based on controls (myotubes exposed to medium from non-transfected HEK293 cells). The size cut-off for a cluster is 2.5 μm^2^, with clusters greater than 2.5 μm^2^ counted.

1. **Statistical analysis**

For expression of mutant and wild type agrin, statistical comparison was performed by two-way ANOVA with multiple comparisons (GraphPad Prism). For AChR clustering assay, data was analyzed using unpaired Student’s t-test (GraphPad Prism). * p < 0.05, ** p < 0.01, *** p < 0.001, **** p < 0.0001.
